# Active surveillance for recurrent low-grade non-muscle-invasive bladder cancer: Can we take any advantage from the COVID-19 crisis?

**DOI:** 10.1080/2090598X.2020.1772031

**Published:** 2020-06-14

**Authors:** Rodolfo Hurle, Carmen Maccagnano

**Affiliations:** Department of Urology, Istituto Clinico Humanitas IRCCS, Clinical and Research Hospital, Rozzano, Italy; Department of Surgery, Division of Urology, Azienda Socio Sanitaria Territoriale Lariana, Nuovo Ospedale Sant’Anna, San Fermo Della Battaglia, Italy

Bladder cancer represents one of the most expensive malignancies to treat and follow-up, due to its high recurrence rate and cancer-specific mortality rate of <1% [1–3].

One of the treatment options for low-grade (LG) non-muscle-invasive bladder cancer (NMIBC) is represented by active surveillance (AS), as recommended by International Guidelines [4–6].

AS was first suggested in 2003 by Soloway and Coll [7], who described it as safe and valid alternative strategy to transurethral resection of bladder tumour (TURBT) for LG-NMIBC, due to minimal risk of progression and impact on cancer-specific survival. In fact, AS allows for a reduction in the number of TURBTs throughout the patient’s lifetime, without compromising the possibility of intervention in case of progression.

Moreover, Soloway and Coll [7] encouraged consideration of the concept of an observational strategy in these low-risk (LR) tumours, considering the fact that a non-negligible number of patients (pts) are old and often present considerable comorbidities. In such pts, partly due to anaesthesia and to complications of TURBT including bleeding (particularly in those pts with anti-coagulants), bladder perforations and urethral strictures, risks increase with the number of repeated procedures, as well as the costs related to hospital stays and pts management.

Thus, subsequent papers reported that experienced clinicians could identify lesions with a LG Ta appearance during cystoscopy with a high degree of accuracy at the pathological examination, especially in case of smaller tumours [8–10]. Therefore, tumour size, haematuria, and cytology status can be used to determine the need for resection.

In 2016 our group published the results of pts with LR NMIBC included in an Italian national observational programme [Bladder Cancer Italian Active Surveillance (BIAS) project] after diagnosis of recurrence [11]. The inclusion and exclusion criteria, as well as the follow-up procedures in our study, are reported in Table 1.

**Table 1.** Inclusion and exclusion criteria, as well as the follow-up protocol, in the Bladder Cancer Italian Active Surveillance (BIAS) project.

Inclusion criteria	Exclusion criteria	Follow-up
1. NMIBC with stage pTa (Grade 1–2) and pT1a (Grade 2; extending into the lamina propria, but above the level of the muscularis mucosa); 2. Single tumour size <10 mm; 3. Number of lesions ranging from one and five; 4. Absence of haematuria; 5. Presence of negative urine cytology; 6. Subscribed informed consent.	1. History of a High-grade carcinoma (Grade 3); 2. History of carcinoma *in situ*; 3. Positive cytology findings have to be excluded.	Urine cytology, together flexible cystoscopy every 3 months during the first year and every 6 months during the subsequent years

In 2018, we reported that the grade and stage progression rate was 13.1% and 7.4%, respectively [12]. Thus, we suggested that NMIBC recurrences with LG appearance, after initial pathological diagnosis of a LG Ta tumour, should appropriately be managed using an AS protocol [13].

Moreover, our group have recently demonstrated that a proportion of pts under AS, operated with TURBT because of ‘typical neoplastic appearance’, showed pathological data that were significantly different from the initial impression, even if given by an expert urologist. As a matter of fact, ~30% of pts were deemed to have AS failure but did not harbour any neoplastic lesion at the final pathological specimen [13]. These data, although surprising, seem to strengthen the role of AS in a selected population with recurrent NMIBC.

In our Centre, about 20–25% of pts with LG Ta recurrence after LR NMIBC meet the inclusion criteria for the BIAS project. Interestingly, pts usually appreciate the opportunity of being monitored with flexible cystoscopies in an outpatient setting every 3–4 months, instead of being operated upon, and no one has dropped out of the protocol.

Considering the economic impact, our recent analysis of resource consumption showed that AS can reduce the life-time cost of pts with small LG pTa NMIBC by avoiding unnecessary frequent surgeries without increasing the risk of progression [12].

Before the coronavirus disease 2019 (COVID-19) outbreak, >200 pts were on AS and we usually performed seven cystoscopies per week in an outpatient setting. However, the pandemic has led to a rapid change in the management of pts with NMIBC, leading to a change of recommendations by scientific societies [14,15].

The number of pts who can potentially meet the inclusion criteria of AS, as well as the possibility to perform follow-up procedures (including urine cytology) has significantly decreased due to restricted access to hospitals.

Regarding cystoscopies, the number of procedures that can be performed daily has been consistently reduced. These limitations may lead to performing an increasing number of TURBTs, potentially putting pts at increased risk, as aforementioned. In this scenario, we can propose two possible amendments of the BIAS project, strictly applicable during this pandemic:

1. An increase in the maximum number of lesions (from five to seven), which leads to TURBT. This suggestion is already desirable considering TURBT itself as an intervention to be postponed in this category of pts [14–16].
2. The follow-up period during AS can be extended from 3 to 6 months.

Curiously, we could take advantage of the COVID-19 pandemic by increasing the number of pts in the AS protocol, due to restricted access both to hospitals and diagnostic procedures in general.
